# Correction: Salimiyekta et al. Random Regression Model for Genetic Evaluation and Early Selection in the Iranian Holstein Population. *Animals* 2021, *11*, 3492

**DOI:** 10.3390/ani12111358

**Published:** 2022-05-26

**Authors:** Yasamin Salimiyekta, Rasoul Vaez-Torshizi, Mokhtar Ali Abbasi, Nasser Emmamjome-Kashan, Mehdi Amin-Afshar, Xiangyu Guo, Just Jensen

**Affiliations:** 1 Department of Animal Science, Science and Research Branch, Islamic Azad University, Tehran 14515-775, Iran; yasamin.sa@qgg.au.dk (Y.S.); nasser_ejk@yahoo.com (N.E.-K.); aminafshar@gmail.com (M.A.-A.); 2 Department of Animal Science, Faculty of Agriculture, Tarbiat Modares University, Tehran 14115-111, Iran; 3 Department of Animal Breeding and Genetics, Animal Science Research Institute of Iran, Karaj 31585-963, Iran; pmaz_abbasi@yahoo.com; 4 Center for Quantitative Genetics and Genomics, Aarhus University, 8830 Tjele, Denmark; xiangyu.guo@qgg.au.dk (X.G.); just.jensen@qgg.au.dk (J.J.)

## Remove One Affiliation from First Author

There was an error in the original publication [1]. The first author (Yasamin Salimiyekta) had two affiliations listed (Azad University and Aarhus University). According to a rule of Azad University, the second affiliation of Yasamin Salimiyekta must be removed and Azad University should be the sole affiliation.
